# Metal Oxide Nanowire-Based Sensor Array for Hydrogen Detection

**DOI:** 10.3390/mi14112124

**Published:** 2023-11-19

**Authors:** Dario Zappa, Navpreet Kaur, Abderrahim Moumen, Elisabetta Comini

**Affiliations:** 1 SENSOR Laboratory, Department of Information Engineering (DII), University of Brescia, Via Valotti 9, 25133 Brescia, Italy; n.kaur001@unibs.it (N.K.); elisabetta.comini@unibs.it (E.C.); 2 Department of Mathematical, Physical and Computer Sciences, University of Parma, Parco Area delle Scienze, 7/A, 43124 Parma, Italy; moumen.abde10@gmail.com; 3 Institute of Materials for Electronics and Magnetism Istituto dei Materiali per l’Elettronica e il Magnetismo (IMEM)—Consiglio Nazionale delle Ricerche (CNR), Parco Area delle Scienze, 37/A, 43124 Parma, Italy

**Keywords:** hydrogen, metal oxide nanowires, chemical sensors

## Abstract

Accurate hydrogen leakage detection is a major requirement for the safe and widespread integration of this fuel in modern energy production devices, such as fuel cells. Quasi-1D nanowires of seven different metal oxides (CuO, WO_3_, Nb-added WO_3_, SnO_2_, ZnO, α-Bi_2_O_3_, NiO) were integrated into a conductometric sensor array to evaluate the hydrogen-sensing performances in the presence of interfering gaseous compounds, namely carbon monoxide, nitrogen dioxide, methane, acetone, and ethanol, at different operating temperatures (200–400 °C). Principal component analysis (PCA) was applied to data extracted from the array, demonstrating the ability to discriminate hydrogen over other interferent compounds. Moreover, a reduced array formed by only five sensors is proposed. This compact array may be easily implementable into artificial olfaction systems used in real hydrogen detection applications.

## 1. Introduction

Industrial and technological developments have resulted in a rapid increase in the rate of global energy consumption, with the energy demand expected to increase even further in the following years also due to the increase in world population [1]. Nowadays, fossil fuel resources are still the dominant source of energy, despite strong attention being devoted to alternative and renewable energy sources. In the past decade, due to the increased use of fossil fuels, the amount of carbon emissions has grown dramatically, combined with an increase in the emission of greenhouse gases (GHGs) like CO_2_, NO_x_, and SO_x_, which contribute to climate change [2,3]. Moreover, with the world’s fossil reserves depleting, it is necessary to move toward a sustainable and environmentally genial source of energy [4]. Recently, the European Commission launched the European Green Deal with the scope of transforming the EU into a modern, resource-efficient, and competitive economy, ensuring carbon neutrality by 2050 [5].

Unfortunately, an efficient and economical route for utilizing renewables, including solar, bio, and wind, as a source for large-scale commercial energy production is not yet available. This gives a significant scope for hydrogen, a clean source of fuel, to be one of the future sources of energy. The combustion of hydrogen produces only water, and advances in hydrogen fuel cell technology can significantly decrease the carbon emissions related to automotive, local power generation, and others [6]. The major drawbacks of hydrogen are that it is an odorless, colorless, and highly dangerous gas not readily available in its elemental form, with lower and upper flammability limits of 4% and 75%, respectively. Therefore, it is necessary to accurately monitor the storage of hydrogen to promptly identify possible leakages.

Nowadays, artificial olfaction systems integrate sensor arrays formed by a combination of different sensing elements based on different materials and working principles, such as conductometric [7,8], piezoelectric [9], field-effect transistors [10], optical sensors [11,12]. Among these, metal oxide (MOX) materials are a class of materials well known in the chemical sensing field, representing the current state-of-the-art technology. These materials have been investigated largely due to their abundance, cheap and easy fabrication, and high performance [13].

In 1991, it was demonstrated for the first time that reducing the size of metal oxide materials to the nanoscale could lead to a substantial enhancement of their sensing properties [14]. Since then, researchers have paid specific attention to the development of novel nanostructures, constantly reducing the grain size to improve the performance of the devices [15]. Among the possible nano-scaled morphologies, metal oxide nanowires are considered one of the most promising for the fabrication of chemical sensors due to their extremely high surface-to-volume ratio, high stability and crystallinity, and unique electrical and chemical properties [16,17,18].

In this work, quasi-1D nanowires of seven different metal oxides (CuO, WO_3_, Nb-added WO_3_, SnO_2_, ZnO, α-Bi_2_O_3_, NiO) were integrated into a conductometric sensor array to evaluate the hydrogen sensing performance in presence of interfering gaseous compounds, namely carbon monoxide, nitrogen dioxide, methane, acetone, and ethanol. Principal component analysis was performed to highlight the properties of the array. Moreover, a reduced array was proposed, selecting the most uncorrelated features of the original array.

## 2. Materials and Methods

### 2.1. Sensors Fabrication

ZnO, WO_3_, SnO_2_, NiO, and α-Bi_2_O_3_ nanowires have been prepared via the evaporation condensation method using the vapor liquid solid (VLS) mechanism, which uses a catalyst to assist the growth of the MOX nanowires and control the diameter of these nanostructures., The growth process mainly consists of the evaporation of a source material (metal or metal oxide powder) followed by the condensation on the substrate previously catalyzed via the specific catalyst. The interaction of the vapor (containing the atoms of the desirable MOX) with the liquid catalyst clusters at high temperatures can be summarized in three steps: nucleation, diffusion, and crystallization.

In our case, ZnO, WO_3_, SnO_2_, NiO, and α-Bi_2_O_3_ nanowires were grown using Au as a catalyst to assist the growth. In particular, these Au catalysts behave as active sites for the nucleation and growth of nanowires.

The noble metal catalyst was deposited via RF magnetron sputtering (Kenotec, Milano, Italy) on alumina substrates (99% purity, 2 × 2 mm^2^, Kyocera, Kyoto, Japan) using an argon plasma at room temperature (duration: 5 s; power: 70 W). Target metal oxide powder was placed in the center of an alumina tubular furnace at a high temperature, while the catalyst substrates were placed in a colder region of the furnace to promote condensation. An inert gas flow (argon) was used to transfer the evaporated material toward the colder region of the furnace to force condensation into the liquid droplets of noble metal catalyst. Detailed information on the synthesis process and mechanism of ZnO [19], WO_3_ [20], SnO_2_ [21], NiO [22], and α-Bi_2_O_3_ [23] nanowires may be found elsewhere.

Instead, CuO and Nb-added WO_3_ nanowires were prepared using a simple thermal oxidation technique in a controlled environment. A thin copper (500 nm) was deposited via RF magnetron sputtering starting from a pure copper target (99.9% purity). For Nb-added WO_3_, we used a pure tungsten target (99.9% purity) with four niobium stubs inserted, resulting in a metal alloy composed of 97% W–3% Nb [24]. In this case, the thickness of the layer was 200 nm.

Samples were placed in the tubular furnace and heated at the required temperature to promote the formation of the nanowires. For thermal oxidation, no catalyst is required as the formation of the nanowires is mainly due to the mechanical stress given by the oxidation process [25].

Further details on the synthesis of CuO [26] and Nb-added WO_3_ [24] nanowires may be found elsewhere. A summary of deposition techniques, experimental conditions and related literature is reported in Table 1.

All metal oxide materials were directly synthetized on 2 × 2 mm^2^ alumina substrate. After the growth of the sensing materials, platinum interdigited electrodes (IDE) and heating elements were deposited on the top and bottom sides of the samples, respectively, via DC magnetron sputtering using the shadow mask technique. More specifically, an adhesion layer of TiW was deposited on top of active materials using a 75 W argon plasma (3 min, 300 °C, 4.5 mTorr), followed by the deposition of the platinum IDE (20 min, 300 °C, 4.5 mTorr, 75 W argon plasma). On the other hand, on the backside of the samples, a TiW adhesion layer followed by the platinum heating element (20 min, 300 °C, 4.5 mTorr, 75 W argon plasma) was deposited. Sensing chips were then mounted on transistor outline (TO) packages using electro-soldered gold wires.

### 2.2. Gas Sensing Measurements

The goal of the present work is to evaluate the hydrogen (H_2_) sensing performance of the fabricated array in presence of interfering gaseous compounds, namely carbon monoxide (CO), nitrogen dioxide (NO_2_), methane (CH_4_), acetone (C_3_H_6_O) and ethanol (C_2_H_5_OH). For this purpose, conductometric sensors were evaluated using a flow-through volt-amperometric technique in a custom measurement chamber.

Sensors were placed in a custom stainless-steel chamber (1 L volume) located inside a climatic chamber fixed at 20 °C to neglect any effect of external temperature variations. A fixed voltage of 1 V was applied to the sensors (Agilent E3631A power supply, Santa Clara, CA, USA), measuring at the same time the electrical conductance of each sensor using dedicated picoammetters (Keithley 6485, Cleveland, OH, USA).

To identify the optimal working conditions of each device, measurements were performed at different temperatures (200 °C, 250 °C, 300 °C, 350 °C, 400 °C), keeping the relative humidity fixed at 50% @ 20 °C, to simulate real-world applications. The temperature of each sensor was controlled independently by modulating the electric power applied to heaters using Thurlbly-Thandar PL330DP power supplies.

Prior to the effective measurements, sensors were thermally stabilized inside the climatic chamber at desired working temperature for 8 h in the presence of a humid air flow of 200 standard cubic centimeters per minute (SCCM). Humidified air was produced by flowing synthetic dry air through a Drechsel bottle in a condensation vessel to favor the condensation of saturated vapor. Test chemical compounds, with a certified composition and concentration, were supplied by SOL (Monza, Italy) and were mixed with dry synthetic air by MKS Instrument (Andover, United States of America) mass flow controllers, maintaining a total flow of 200 SCCM.

Table 2 reports the gas concentration injected in the chamber for each target compound. After the 30 min exposure to the selected gas concentration, synthetic air flow was restored for 60 min to allow the recovery of the electrical conductance baseline.

The response of each sensor is calculated as the ratio between the variation of the electrical conductance over the baseline (ΔG/G), using the following formulas for reducing and oxidizing gases, respectively, for n-type materials:

Reducing compounds: Response = (G_gas_ − G_air_)/G_air_

Oxidizing compounds: Response = (G_air_ − G_gas_)/G_gas_

where G_air_ and G_gas_ are, respectively, the sensor conductance of the baseline and in presence of target gaseous compound. For p-type materials (α-Bi_2_O_3_, CuO, and NiO), these formulas are swapped.

## 3. Experimental Results

### 3.1. Materials Characterization

**Figure 1:**
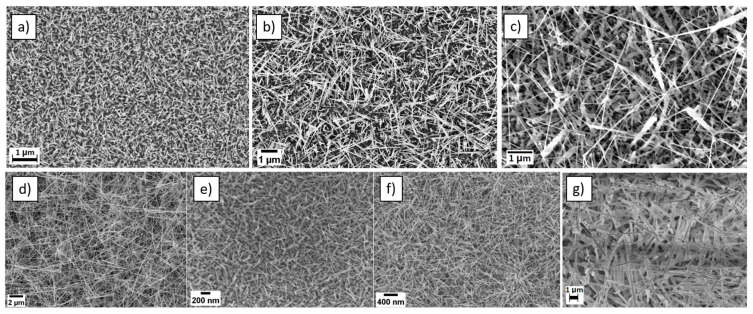
SEM pictures of ZnO (**a**), CuO (**b**), NiO (**c**), SnO_2_ (**d**), WO_3_ (**e**), Nb-added WO_3_ (**f**), and α-Bi_2_O_3_ (**g**) nanowires.

Figure 1 shows the SEM images of ZnO, WO_3_, SnO_2_, NiO, Nb-added WO_3_, CuO and α-Bi_2_O_3_ nanowires. All materials show a scattered and homogenous nanowire morphology with high density and high aspect ratio.

α-Bi_2_O_3_ is a p-type semiconductor that has a monoclinic crystalline structure with space group symmetry P21/c [23], while n-type ZnO nanowires have a hexagonal crystalline structure with space group symmetry P63mc [19].

Specifically, long ZnO nanowires (around 700 nm in length) with small diameters (around 25 nm) have been grown, while α-Bi_2_O_3_ nanowires have a bigger diameter (around 100 nm) but they are long (around 6 µm in length). Most importantly, the presence of gold nanoparticles at the tip of nanowires in α-Bi_2_O_3_ nanowires confirms the VLS mechanism. It has been reported that gold nanoparticles could contribute to enhancing the gas sensing performance due to the spillover effect [23].

P-type NiO nanowires have a cubic crystallographic structure with an Fm-3m space group. The diameter of the nanowires was in the range of 20 to 60 nm and a length at a micrometer scale [22]. N-type SnO_2_ nanowires exhibit morphology similar to NiO one but have a tetragonal crystal structure with a P42/mnm space group [21]. Instead, n-type WO_3_ nanowires possess a diameter in the range of 10–30 nm and a length under 100 nm, with a monoclinic crystallographic structure belonging to the P2/m space group [20].

On the other hand, for thermally oxidized nanowires, Nb-added WO_3_ have the same monoclinic structure and aspect ratio as pristine WO_3_ nanowires, as the amount of niobium added does not significantly influence their crystal structure and morphology [24]. Moreover, this material exhibits an n-type behavior like pristine WO_3_. CuO, instead, is a p-type material that has a monoclinic structure with C2/c with an average length of a few micrometers [26].

### 3.2. Gas Sensing Performances

**Figure 2:**
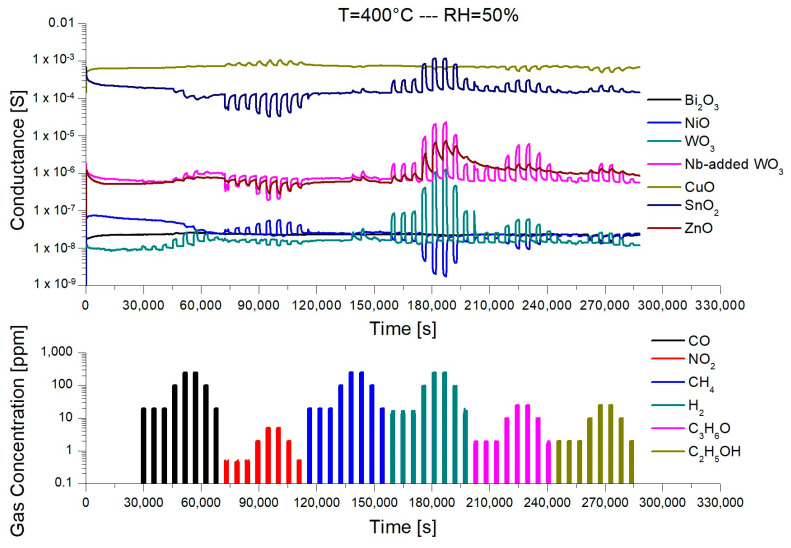
Dynamic response of the seven different sensors in presence of various gaseous compounds (CO, NO_2_, CH_4_, H_2_, C_3_H_6_O, and C_2_H_5_OH) at 400 °C and relative humidity (RH) of 50% @ 20 °C.

A typical dynamic response of the sensors at 400 °C is reported in Figure 2, reporting the electrical conductance of each sensor overtime during the injections of the different chemical compounds. At this temperature, most of the sensors exhibit a strong variation of the conductance in the presence of hydrogen, which is the main target compound of the present study. However, these sensors also respond to other compounds, mainly nitrogen dioxide, acetone, and ethanol. Interestingly, CuO and α-Bi_2_O_3_ devices, despite a low overall response, do not have any appreciable response to hydrogen but only to the identified interfering gases. This is an important feature to enhance the selectivity of the array, as described afterward.

From Figure 2, it is possible to observe that ZnO is not recovering the baseline properly. This behavior has been observed for all temperatures investigated and could lead to some issues in case it is selected and used in the final array. Moreover, all sensors have a very low response to methane, which affects the electrical conductance of the nanowires only at concentrations higher than 100 ppm.

**Figure 3:**
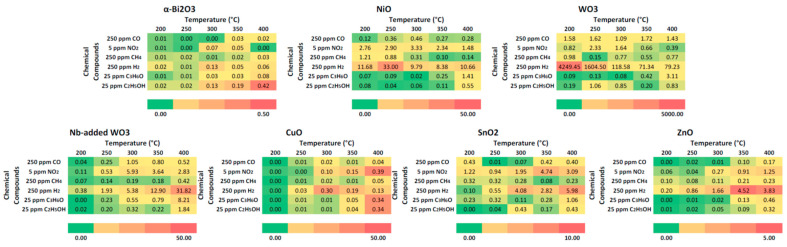
Response of the sensors array to specific concentrations of chemicals, calculated as ΔG/G (mean values of multiple measures). Higher numbers (in red) are better.

Figure 3 reports the heatmaps of sensors’ response at different concentrations of chemical compounds in the 200–400 °C temperature range. Sensor responses have been calculated as ΔG/G, and reported numbers are the mean values of multiple measurements. Each sensing material has its own response spectrum, which is influenced by the operating temperature. Humidity in the environment often is considered an interfering compound and should be treated as other chemicals. However, at this stage, the relative humidity during all measurements was kept constant at 50%.

α-Bi_2_O_3_ nanowires are not sensitive to hydrogen at the investigated temperature. However, at 400 °C, this sensor is very selective to ethanol (C_2_H_5_OH) and partially to acetone (C_3_H_6_O). Similar behavior is observed in the case of the CuO sensor at 400 °C, which is selective to ethanol, acetone, and nitrogen dioxide. Despite the values of calculated responses being relatively small, these sensors provide complementary information that is fundamental to increasing the selectivity of the array.

NiO device is moderately sensitive to hydrogen at every temperature, exhibiting the maximum response at 250 °C with a partial cross-sensitivity with nitrogen dioxide. This cross-sensitivity is lower at 250 °C compared to other temperatures.

The most sensitive material to hydrogen is pristine WO_3_, which has a huge response to 250 ppm at 200 °C. Interestingly, increasing the operating temperature leads to faster response and recovery times but decreases the response of the device. Nevertheless, the response and the selectivity of this material are still good even at 400 °C. Nb-added WO_3_ has a completely different behavior, as the optimal response to hydrogen is obtained at 400 °C but with a partial cross-sensitivity to acetone.

ZnO and SnO_2_ have similar behavior, with a maximum response to hydrogen at high temperatures (350 °C and 400 °C, respectively) but with a significant cross-sensitivity to nitrogen dioxide and acetone.

We compared the performance of developed nanowire-based sensors with some commercial devices. TGS 2616-C00 by Figaro (Osaka, Japan) is a device that is specifically designed for hydrogen sensing [27]. According to sensor specifications, the response to 250 ppm of hydrogen is ≈25, lower than the proposed WO_3_-nanowire sensor. GMV-2021B, a MEMS hydrogen sensor developed by Zhengzhou Winsen Electronic Technology Co., Ltd. (Zhengzhou, China), has a response of ≈10 to the same concentration of hydrogen while exhibiting some cross-interference to carbon monoxide [28]. The performance of this device is lower compared to WO_3_, Nb-WO_3_, and NiO. MiCS-5524 from SGX Sensortech (Neuchatel, Switzerland) is a compact MOS sensor for indoor carbon monoxide and natural gas leakage detection [29]. Despite having a good response of >30 to 250 ppm, it has a huge cross-sensitivity to carbon monoxide and ethanol. Finally, GGS 6530 T from UST Umweltsensortechnik GmbH (Geratal, Germany) has a response of ≈10 but is also sensitive to methane [30].

Response and recovery times are calculated as the time required by each sensor to reach 90% of the response and 90% of the baseline, respectively. The measurement chamber used in our experimental setup has a volume of 1 L to host multiple sensing devices simultaneously. However, using a 200 sccm flow, it takes 3–5 min to completely fill or empty the chamber. According to the performed measurements, recorded response and recovery times at temperatures higher than 300 °C are always comparable with the chamber filling time, with the sole exception of ZnO, as seen in Figure 2. Therefore, we are not able to estimate properly the kinetics of the surface reactions. For this purpose, we rely only on the electrical response for the identification of the features of the principal component analysis (PCA) introduced in the next section.

### 3.3. Data Analysis

Collected data were analyzed with chemometrics tools based on multivariate statistics, i.e., principal component analysis (PCA). PCA is a simple, non-parametric method for extracting relevant information from confusing or complex data sets, such as the one coming from multiple sensors. It is a powerful tool to reduce complex data sets to lower dimensions to extract hidden or simplified information [31].

For the calculation of the principal components, we selected the response of each device at every temperature as a feature. However, we removed from the dataset the measures at 200 °C due to the slow kinetics of the sensors and the overall small responses (except pristine WO_3_), which can cause problems in the identification of the compounds. Therefore, we defined 48 features to be used in the PCA.

**Figure 4:**
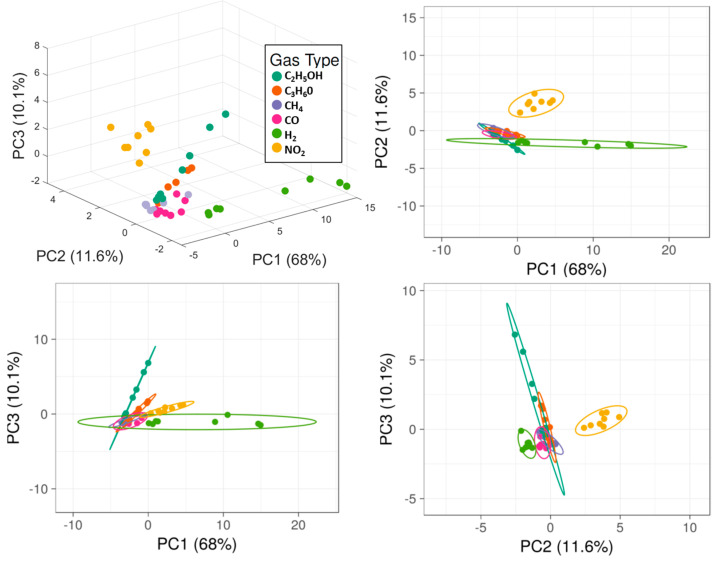
PCA applied to data from all sensors working in 250–400 °C temperature range. Axes report the principal components 1, 2, and 3 that explain 68%, 11.6%, and 10.1% of the total variance, respectively. Prediction ellipses are such that with a probability of 0.95, a new observation from the same group will fall inside the ellipse.

We used ClustVis and Mathworks MATLAB R2021a software to elaborate the data [32,33]. Original values are ln(x + 1)-transformed to reduce numerical spread as data from sensors are distributed over multiple orders of magnitude. Unit variance scaling is applied to rows, while singular value decomposition (SVD) with imputation is used to calculate principal components. Figure 4 reports the first free principal components in 3D and their respective projections in 2D space.

**Figure 5:**
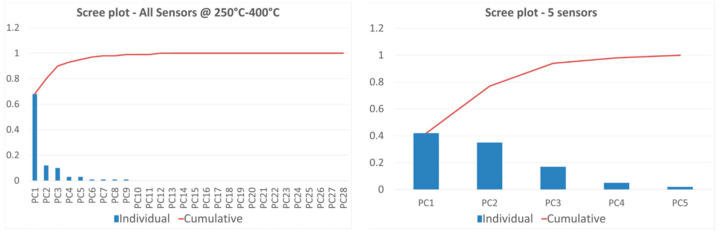
Scree plots of the individual (in blue) and cumulative (in red) variance carried in principal components calculated using all sensors at 250–400 °C (**Left**) and using the reduced array (**Right**).

The calculated PCA in three dimensions takes more than 90% of the total variance of the data (Figure 5, Left). Data points related to hydrogen (highly correlated to PC1), nitrogen dioxide, acetone, and ethanol (highly correlated to PC3) are clearly distinguishable, being fuzzy only at the lowest concentration tested. It is not possible to easily detect carbon monoxide and methane with the proposed array, but this hardly affects hydrogen recognition. A quantitative estimation of the presence of hydrogen is even possible, as data points from the same concentration are close and isolated from others.

Despite the promising performance of the array, the number of sensors required (28, operating at different temperatures) limits its use in real applications. Moreover, it is observed that there is a sort of correlation among data obtained from different materials or data obtained from the same material but at different temperatures, as seen in the heatmaps of Figure 3. By calculating the Pearson correlation coefficients extracted from the features data it is evident that many features (the sensors) carry redundant information. This is confirmed also by checking the PCA loadings: many features have similar loadings on the first three principal components. Therefore, some of the features may be safely disregarded without any appreciable loss of performance. Recursively removing the features exhibiting the higher correlation to the others, we reduced the array to only five different sensors: α-Bi_2_O_3_ @ 400 °C, SnO_2_ @ 350 °C, WO_3_ @ 300 °C, CuO @ 250 °C and SnO_2_ @ 250 °C.

**Figure 6:**
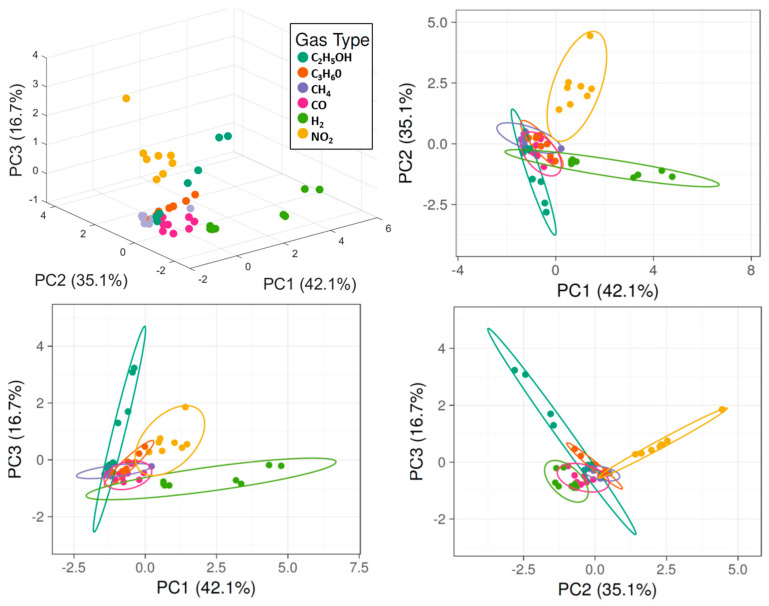
PCA applied to data from α-Bi_2_O_3_ @ 400 °C, SnO_2_ @ 350 °C, WO_3_ @ 300 °C, CuO @ 250 °C, and SnO_2_ @ 250 °C. Axes report the principal components 1, 2, and 3 that explain 42%, 35.1%, and 16.7% of the total variance, respectively. Prediction ellipses are such that with a probability of 0.95, a new observation from the same group will fall inside the ellipse.

As performed for the original array, values are ln(x + 1)-transformed, unit variance scaling is applied to rows, while singular value decomposition (SVD) with imputation is used to calculate principal components. The selection of the first three components includes 94% of the original variance (Figure 5, Right). PCA plots of the five-sensors array reported in Figure 6 resemble the ones extracted from the original and complete array (Figure 4). Differences are related to the loss of information. Hydrogen is still bound to PC1, while ethanol is bound to PC3, as in the original array. The reduced array allows the discrimination of hydrogen, ethanol, and nitrogen dioxide but loses the ability to distinguish acetone as data points are too close to other gases. However, detection and quantification of hydrogen, which was the main target of this work, is still possible.

Further reducing the number of features (sensors) is possible, such as removing WO_3_ @ 300 °C. However, this is not recommended as hydrogen data points at low concentrations start to overlap those of other compounds, limiting the performance of the array.

## 4. Conclusions and Future Perspectives

In this work, quasi-1D nanowires of seven different metal oxides (CuO, WO_3_, Nb-added WO_3_, SnO_2_, ZnO, α-Bi_2_O_3_, NiO), synthesized via evaporation condensation and thermal oxidation techniques, were integrated into a conductometric sensor array. Accurate gas sensing measurements in a controlled environment allow us to evaluate the hydrogen sensing performances in the presence of possible interfering compounds, namely carbon monoxide, nitrogen dioxide, methane, acetone, and ethanol, at different operating temperatures (200–400 °C).

Principal component analysis (PCA) was applied to data extracted from the array, demonstrating the ability to discriminate hydrogen over interferent compounds but also a strong redundancy and correlation of the different sensors operated at various temperatures. Therefore, we selected the most promising materials to be included in a revised and compact array composed of only five sensors: α-Bi_2_O_3_ @ 400 °C, SnO_2_ @ 350 °C, WO_3_ @ 300 °C, CuO @ 250 °C, and SnO_2_ @ 250 °C. According to PCA, the discrimination capabilities of the reduced array were similar to the original ones. However, for the precise assessment of the hydrogen sensing performance, multiple classification methods should be implemented and evaluated, such as learning vector quantization (LVQ) neural network [34], partial least squares discriminant analysis (PLS-DA) or nearest neighbor (k-NN) [35] or artificial neural networks (ANNs) [36]. Nevertheless, the proposed compact array is suitable to be implemented into artificial olfaction systems (e-noses) used in real hydrogen detection applications.

## Figures and Tables

**Table 1:** Synthesis parameters used for the fabrication of the active sensing materials.

Material	Technique	Powder Temperature	Substrate Temperature	Time	Pressure	Atmospheric Conditions	Catalyst	Reference
ZnO	Evaporation-condensation	1200 °C	500 °C	15 min	10 mbar	Argon flow: 75 sccm	Au	[19]
α-Bi_2_O_3_	Evaporation-condensation	1000 °C	500 °C	10 min	10 mbar	Argon flow: 75 sccm	Au	[23]
NiO	Evaporation-condensation	1450 °C	930 °C	12 min	1 mbar	Argon flow: 100 sccm	Au	[22]
SnO_2_	Evaporation-condensation	1370 °C	860 °C	2 min	10 mbar	Argon flow: 100 sccm	Au	[21]
WO_3_	Evaporation-condensation	1100 °C	525 °C	15 min	1 mbar	Argon flow: 100 sccm	Au	[20]
Nb-WO_3_	Thermal oxidation	-	600 °C	1 h	1 mbar	Argon flow: 10 sccm	-	[24]
CuO	Thermal oxidation	-	400 °C	4 h	1000 mbar	-	-	[26]

**Table 2:** Chemical compounds investigated and relative concentrations injected into the chamber.

Gas Type	Concentrations Injected in Chamber (ppm)
CO	20, 20, 20, 100, 250, 250, 100, 20
NO_2_	0.5, 0.5, 0.5, 2, 5, 5, 2, 0.5
CH_4_	20, 20, 20, 100, 250, 250, 100, 20
H_2_	15, 15, 15, 100, 250, 250, 100, 15
C_3_H_6_O	2, 2, 2, 10, 25, 25, 10, 2
C_2_H_5_OH	2, 2, 2, 10, 25, 25, 10, 2

## Data Availability

The data presented in this study are available upon request from the corresponding author.
